# Allosteric Activation of GDH/TCA Pathway Reduces Pathological Build-Up and Promotes Neuronal Survival in an In Vitro Model of Alzheimer’s Disease

**DOI:** 10.3390/biom16050667

**Published:** 2026-04-30

**Authors:** Tiziano Serfilippi, Silvia Piccirillo, Alessandra Preziuso, Valentina Terenzi, Raffaella Ciancio, Simona Magi, Vincenzo Lariccia, Agnese Secondo

**Affiliations:** 1 Department of Biomedical Sciences and Public Health, School of Medicine, University “Politecnica delle Marche”, Via Tronto 10/A, 60126 Ancona, Italy; t.serfilippi@pm.univpm.it (T.S.); s.piccirillo@staff.univpm.it (S.P.); a.preziuso@staff.univpm.it (A.P.); v.terenzi@pm.univpm.it (V.T.); s.magi@staff.univpm.it (S.M.); 2 Division of Pharmacology, Department of Neuroscience, Reproductive and Odontostomatological Sciences, School of Medicine, University of Naples “Federico II”, Via S. Pansini 5, 80131 Naples, Italy; raffaella.ciancio@unina.it

**Keywords:** Alzheimer’s disease, neuronal cells, glutamate dehydrogenase, BCH, oxidative stress, organellar calcium dyshomeostasis, mitochondria, phosphorylated tau, amyloid beta

## Abstract

Mitochondrial dysfunction is a relevant hallmark of Alzheimer’s disease (AD), contributing to the impaired metabolic homeostasis involved in neuronal loss and cognitive decline. In this study, we target the metabolic dysfunction occurring in AD through a novel pharmacological approach involving the modulation of glutamate dehydrogenase (GDH), which converts glutamate to α-ketoglutarate and supports the tricarboxylic acid (TCA) cycle. In our experimental models (i.e., differentiated SH-SY5Y cells and primary rat cortical neurons exposed to glyceraldehyde and amyloid-beta peptide 1-42, respectively), the allosteric GDH activator 2-Aminobicyclo-(2,2,1)-heptane-2-carboxylic acid (BCH) increased mitochondrial ATP production, improved cellular bioenergetics, and reduced oxidative stress, ultimately promoting neuronal survival. Ionic dysfunctions in AD are linked to disrupted calcium homeostasis and organelle storing properties. In this context, GDH activation potentiated mitochondrial and endoplasmic reticulum calcium buffering capacity by enhancing store-operated calcium entry. Oxidative stress, largely driven by mitochondrial ROS overproduction, represents another major contributor to AD pathology. In our AD models BCH-mediated GDH activation reduced ROS formation and restored mitochondrial membrane potential (ΔΨ_m_). Importantly, these metabolic and ionic improvements were associated with decreased accumulation of amyloid-β (Aβ_1-42_) and phosphorylated tau (pTau), two key AD biomarkers. Overall, modulation of the GDH/TCA pathway represents a promising approach for restoring metabolic dysfunctions and counteracting oxidative stress and ionic dysregulation and therefore AD neurodegeneration.

## 1. Introduction

Alzheimer’s disease is a progressive neurodegenerative disease and the most common type of dementia [1], estimated to affect over 55 million people worldwide, a number that is predicted to rise to 139 million by 2050 [2], mainly due to the ageing of the global population and its growth [3].

Several observational studies have identified a number of potential risk factors [4], many of which relate—directly or indirectly—to energy metabolism, such as obesity [5,6,7], metabolic syndrome, hyperlipidemia and diabetes [5,8]; additionally, statin users have a lower incidence of AD [9,10,11].

However, growing evidence indicates that metabolic alterations emerge very early in AD pathogenesis—even decades before the onset of clinical symptoms; and while cognitive decline directly correlates with glucose metabolism [12], AD neurons show significant impairments in the consumption of glucose, lactate and ketone bodies, since they undergo a reduction of monocarboxylate transporter (MCTs) expression, which is essential for the transport of both ketone bodies and lactate. The same effect occurs for GLUTs, resulting in a decreased glucose uptake, as was evidenced by FDG-PET studies [13,14].

In this framework, AD could be considered as a metabolic disease in which energy failure, oxidative damage, and protein accumulation are intimately connected and cumulatively disrupt the integrity and computational abilities of the brain when the bioenergetic support is not optimized to neuronal needs [15,16].

Indeed, the brain is in a constant state of high energy demand, which is supplied by ATP production, mainly via glycolysis, the tricarboxylic acid (TCA) cycle and oxidative phosphorylation (OXPHOS) [13,17,18]. During OXPHOS, the final acceptor of electrons is oxygen, but its partial reduction may also occur, thereby leading to the formation of mitochondrial ROS [19,20]. Under physiological conditions, ROS act as signaling molecules and regulate transcription pathways. But if they exceed the antioxidant capabilities, they attack cellular biological macromolecules, resulting in oxidative damage, and oxidative stress subsequently sets in, eventually leading to cellular and mitochondrial dysfunction and, finally, apoptosis [15,21]. Mitochondria are not only highly susceptible to ROS, but they are also considered their main producers, especially in the AD brain, where increased oxidative stress is commonly observed [19]. Indeed, mitochondrial deposition of Amyloid beta (Aβ) disrupts the activity of the electron transport chain (ETC), leading to an increase in ROS production, which in turn stimulates the expression of APP while promoting BACE1 enzymatic function, shifting the processing of APP to the amyloidogenic pathway and ultimately promoting Aβ aggregation [21].

Moreover, Aβ oligomers are capable of forming Ca^2+^ permeable pores in the cellular membranes [22,23] and interacting with NMDA receptors, thus activating these receptors and, therefore, increasing the calcium conductance of these receptors. Moreover they are able to bind to AMPA receptors, making these receptors Ca^2+^ permeable [23,24]. The disruption of calcium homeostasis appears to be an early event in the pathophysiological history of the disease [25]. Specifically, calcium overload in the ER is a major pathological finding in AD neurons, which are characterized by an enhanced Ca^2+^ efflux from the ER and a reduced Ca^2+^ influx through SOCE [24,26]. Furthermore, the interplay among lysosomes and other calcium-storing organelles is severely impaired in many neurodegenerative conditions, such as AD [27,28,29].

The remodeling of intracellular calcium dynamics in AD affects the mitochondria as well. Indeed, the increase in cytosolic Ca^2+^ concentration and ER Ca^2+^ release are initially counteracted via an enhanced uptake by mitochondria. Excessive Ca^2+^ levels in the mitochondrial matrix cause a ROS overproduction, damaging biomolecules and the mitochondria as a whole. Furthermore, Aβ promotes the opening of the mitochondrial permeability transition pores (mPTP), a Ca^2+^-dependent channel, eventually leading to a massive efflux of mitochondrial Ca^2+^ into the cytosol and depolarizing the mitochondrial inner membrane. This leads to mitochondrial swelling and loss of membrane stability, releasing mitochondrial proteins such as cytochrome c, which ultimately leads to neuronal cell death, or apoptosis [24].

However, glucose and ketone bodies are not the only energy substrates available to neurons, as neurons possess a diverse battery of bioenergetic pathways [13,17,18]. Specifically, glutamate, which fills a vast range of roles in the CNS, as it is one of the main neurotransmitters and a substrate for the synthesis of proteins and other neurotransmitters, can also be used as a metabolic intermediate for bioenergetic needs [30].

Notably, neurons lack pyruvate carboxylase (PC) and are thereby unable to synthesize glutamate de novo from glucose and instead rely on the glutamate/GABA-glutamine cycle. Glutamate released in the synapsis is taken up by the surrounding astrocytes and converted into glutamine by the glutamine synthetase, an enzyme typically expressed in astrocytes [31,32]. However, neurons can interconvert glutamate and α-ketoglutarate, thanks to the activity of glutamate dehydrogenase (GDH), an enzyme located in the mitochondrial matrix of both neurons and astrocytes, and capable of catalyzing the NAD(P)^+^-dependent oxidative deamination of glutamate to α-ketoglutarate [31], thereby converting glutamate into α-ketoglutarate and ammonia while reducing NAD(P)^+^ to NAD(P)H [33]. Two distinct GDH isoenzymes have been identified so far: GDH1, which is a “housekeeping” enzyme found in most mammals, and GDH2, which is a more recent evolutionary addition found specifically in humans and apes [33]. Both of these are expressed in the mitochondria of cortical astrocytes, while oligodendrocytes only express GDH1. Conversely, GDH2 is found in cytoplasmic structures (which resemble mitochondria) scattered throughout the cell body and the axon, specifically in large cortical neurons with a pyramidal morphology. GDH2 is also observed in the nuclear membrane of small, round neurons [33].

GDH is allosterically regulated by a vast range of different compounds, encompassing, but not limited to, ADP, GTP, NADH, L-leucine, palmityl CoA, spermidine, steroid hormones, neuroleptic drugs and the green tea polyphenol epigallocatechin gallate [34]. Despite their high level of homology, the biochemical, functional and regulatory differences between GDH1 and GDH2 in humans have been characterized; most of these findings are reported in Table 1. In particular, GDH1 is highly inhibited by GTP, whereas GDH2 is resistant to GTP inhibition. GDH2 is more strongly activated by ADP and leucine, compared to GDH1. GDH2 is significantly more sensitive (about 20-fold) to inhibition by 17β-estradiol, compared to GDH1. GDH2 exhibits lower basal activity and greater sensitivity to allosteric regulators, compared to the higher baseline activity of GDH1 [33,35].

Given that the vast majority of relevant drugs are used to treat the symptoms and conditions associated with AD [36,37], and that all currently available disease-modifying treatments can only moderately slow disease progression, but none can halt it or reverse it [38], we need a different therapeutic approach to this devastating disease. With this work, we aim to feed the TCA cycle with α-ketoglutarate derived from glutamate in order to foster mitochondrial ATP synthesis and, thereby, improve the evolution of the cell damage in a chemically induced AD-like model. To this aim, the stimulation of the GDH activity has been achieved via administration of 2-Aminobicyclo-(2,2,1)-heptane-2-carboxylic acid (BCH), a non-metabolized analogue of leucine that acts as a strong allosteric activator of GDH [30,39,40].

**Table 1:** Biochemical and pharmacological features of GDH1 and GDH2.

Biochemical Features	GDH1	GDH2	Notes	References
Basal activity	High	Low	-	[41,42,43,44]
GTP inhibition	Sensitive	Resistant	-	[41,42,43,44]
ADP activation	Moderate	High	-	[42,43]
Leucine activation	Moderate	High	-	[43,44]
Sensitivity to estrogen inhibition	Lower	Higher	-	[45]
Main localization	General/liver	Brain/testis/kidney	-	[46]
Optimal pH	7.75–8.0	7.5	-	[41,44]
Thermal stability	Heat-stable	Thermolabile	-	[41,42]
Allosteric Activation by BCH (2-Aminobicyclo-(2,2,1)-heptane-2-carboxylic acid)	+	- No evidence - No comparative studies	Non-metabolized leucine analogue	[40]
Allosteric Activation by 75-E10 (N1-[4-(2-aminopyrimidin4-yl)phenyl]-3-(trifluoromethyl)benzene-1-sulfonamide	+	- No evidence - No comparative studies	Alleviates GTP inhibitionbinding to the ADP site and causing similar activation	[47]

## 2. Material and Methods

### 2.1. Drugs and Chemicals

The SH-SY5Y cell line was purchased from the American Type Culture Collection (CRL-2266). MEM/F12, fetal bovine serum (FBS), Horse Serum (HS), MitoTracker CM-H2XRos, MitoTracker Red CMXRos and Fluo-4-AM were purchased from Invitrogen Life Technologies (Carlsbad, CA, USA). Lactate dehydrogenase (LDH) reaction mix was purchased from Roche Diagnostics (Monza, Italy). ATPlite was purchased from Revvity (Waltham, MA, USA). The Bio-Rad protein assay dye reagent used for the Bradford Assay was purchased from Bio-Rad (Hercules, CA, USA). Tetramethylrhodamine ethyl ester (TMRE) and Rhod-2-AM were purchased from Abcam (Cambridge, UK). Anti-Aβ_1-42_ mouse monoclonal IgG1 antibody (clone 12F4, Cat. 805501, dilution 1:100 in PBS with 1% BSA for immucytochemistry and clone 6E10 dilution 1:500 in 5% milk for WB) was purchased from Biolegend (San Diego, CA, USA), while anti-PHF-Tau monoclonal IgG antibody (clone AT100, Cat. MN1060, dilution 1:1000 in PBS with 1% BSA) recognizing Thr212 and Ser214, the secondary antibody (Anti-mouse Alexa Fluor 488 dye-conjugated antibody, dilution 1:200, Cat. A110599) and Hoechst 33342 were purchased from Thermo Scientific (Milano, Italy). Dulbecco’s Modified Eagle Medium (DMEM), fetal bovine serum (FBS), penicillin, and streptomycin were purchased from Corning (New York, NY, USA). Calcium ionophore A23187 was purchased from Santa Cruz Biotechnology, Inc. (Dallas, TX, USA). Aβ 1-42 (cd 4014447) was purchased from BACHEM (Bubendorf, Switzerland). All the other chemicals were of analytical grade and were obtained from Sigma-Aldrich (Merck Millipore, Milan, Italy).

### 2.2. Cellular Cultures

#### 2.2.1. SH-SY5Y

The SH-SY5Y cells, a line derived from human neuroblastoma, were cultivated in Dulbecco’s Modified Eagle Medium (DMEM) supplemented with 10% fetal bovine serum (FBS), 100 U/mL penicillin, and 100 µg/mL streptomycin. They were maintained in an incubator at 37 °C with a 5% CO_2_ atmosphere and the cell culture medium was replaced every 2 days. In order to induce their differentiation into neuron-like cells, they were exposed to 10 µM all-trans retinoic acid (RA) for 5 days [48,49].

#### 2.2.2. Rat Primary Cortical Neurons

Mixed cultures of cortical neurons from Wistar rat pups (P1–P4) (Cat. 003WISTAR) were prepared as previously described in prior work [50,51]. Briefly, dissection and dissociation were performed in Ca^2+^/Mg^2+^-free phosphate-buffered saline (PBS) containing glucose (5.5 mM). Tissues were incubated with papain and DNAse (10 min/37 °C). Cells were plated at 0.8–1 × 10^6^ in 12 plastic multiwells pre-coated with poly-D-lysine (10 μg/mL) in MEM/F12—containing glucose, 5% deactivated fetal bovine serum (FBS) and 5% horse serum, glutamine and pen/strep—or at 1.8 × 10^6^ on 25 mm glass coverslips pre-coated with poly-D-lysine (10 μg/mL). Cultures were kept at 37 °C in a humidified atmosphere of 5% CO_2_ and 95% air, and the medium was changed once a week, while experiments were performed between 7 and 10 DIV (days in vitro).

The use of animals and the associated procedures used to isolate cortical neurons were in full compliance with the Ethics Committee for Animal Experiments of the University “Politecnica delle Marche” and in strict accordance with the guidelines of the Italian Ministry of Health (D.L. 26/2014).

### 2.3. Alzheimer’s Disease Experimental Models

In order to induce Alzheimer’s disease alterations in our neuron-like model, differentiated SH-SY5Y cells or primary rat cortical neurons were exposed to glyceraldehyde (GA) 1 mM for 24 h. GA exposure recapitulates fundamental molecular features of AD, and is able to induce the transductional cascades and pathomechanisms found in several AD models [49,52,53].

GA is naturally present in cells and can originate via three pathways: (i) during glycolysis, glucose is converted into glyceraldehyde-3-phosphate and subsequently non-enzymatically dephosphorylated; (ii) in fructolysis, fructokinase and aldolase B convert fructose into GA; and (iii) under hyperglycemia, glucose is transformed into fructose and then GA through the polyol pathway by aldose reductase and sorbitol dehydrogenase [54].

However, GA is normally maintained at low concentrations via its conversion to glyceraldehyde 3-phosphate by glyceraldehyde-3 phosphate dehydrogenase (GAPDH) [55]. Interestingly, GA is capable of inhibiting GAPDH and thereby generating a feed-forward cycle in which the inhibition of GAPDH stimulates the production of GA, leading to the reduced activity of GAPDH. Moreover, GA also proved to be detrimental with respect to the mitochondria [56,57].

To strengthen the translational significance of the study, amyloid-beta peptide1-42 was prepared according to the manufacturer’s instructions and applied to the cells at a final concentration of 5 µM for 48 h to induce Alzheimer’s disease–like cellular alterations.

### 2.4. Viability Assays

#### 2.4.1. LDH Assay

Cell viability was tested by measuring the activity of LDH released into the culture supernatant. Briefly, at the end of each experiment, 50 µL of supernatant from the tested wells was incubated with 50 µL of reaction mix (30 min/RT in darkness). Subsequently, the absorbance was measured at a wavelength of 490 nm with a Victor Multilabel Counter plate reader (Perkin Elmer, Waltham, MA, USA). Values are expressed as a percentage relative to the control [58].

#### 2.4.2. Mitochondrial Activity (MTT Assay)

The MTT assay is a colorimetric detection technique capable of indirectly assessing cell viability through the evaluation of the cell’s capability to reduce the yellow tetrazolium salt 3-(4,5-dimethylthiazol-2-yl)-2,5-diphenyltetrazolium bromide (MTT) to insoluble purple crystals of formazan. At the end of the experimental protocol, the cells were incubated with MTT solution (0.5 mg/mL in PBS) in a humidified incubator (1 h/37 °C in the dark, and in a 5% CO_2_ atmosphere). Subsequently, the supernatant was discarded and the produced formazan crystals were dissolved in DMSO. The absorbance was read at a wavelength of 540 nm using a Victor Multilabel Counter plate reader (Perkin Elmer, Waltham, MA, USA). Values are expressed as a percentage relative to the control [59,60].

### 2.5. ATP Assay

ATP cellular content was evaluated using a commercially available luciferin–luciferase bioluminescent assay (ATPlite, Revvity, Waltham, MA, USA). SH-SY5Y cells were plated and differentiated onto 96-wells View Plates (Revvity, Waltham, MA, USA), while primary rat cortical neurons were plated on 12-wells plates. Afterwards, cells were subjected to the experimental protocol. Later, the assay was performed as per the manufacturer’s instructions. The count per second (CPS) values were quantified utilizing a luminescence-based Victor Multilabel Counter plate reader (Perkin Elmer, Waltham, MA, USA). The measured ATP cellular content was also normalized to the protein content in each well, as measured using the Bradford Assay (Bio-Rad protein assay dye reagent). Values are expressed as a percentage relative to the control [61,62].

### 2.6. Western Blot

Proteins contained in total lysates were separated via electrophoresis on an 8–15% SDS-polyacrylamide gel. Then, nitrocellulose membranes were subjected to the blocking phase (1 h in 5% milk for Amyloid-beta and 3% BSA for pTau) and exposed to the appropriate primary antibody (1:1000 pTau AT100 Thr212, Ser214 Thermo Fisher, Waltham, MA, USA; 1:500 Amyloid-beta clone 6E10 Biolegend/4 °C, O/N). After the exposure to the secondary antibody, blots were developed using an enhanced chemiluminescence detection kit (SupersignalTM West Atto Ultimate Sensitivity Substrate, Thermo Fisher, Waltham, MA, USA), and images were captured and analyzed with Uvitec Nine Alliance analysis software version Q9 Alliance, using a Uvitec Cambridge Chemiluminescence Imaging System (Cambridge, UK).

### 2.7. Mitochondrial ROS Production in Single Cells

Mitochondrial ROS production was quantified using MitoTracker CM-H2XRos. SH-SY5Y cells were plated onto 25 mm coverslips and differentiated using RA. After differentiation, cells were subjected to the experimental protocol.

Subsequently, cells were loaded with MitoTracker CM-H2XRos, with a concentration of 300 nM (30 min/37 °C). Confocal images were obtained using a 510 LSM microscope equipped with a META detection system (Carl Zeiss, Milan, Italy). For visualization of MitoTracker CM-H2XRos, the dye was excited at 543 nm, and its emission was measured at 620 nm.

Images were captured at 5 s intervals, and the basal fluorescence values were monitored for approximately 15 s. At least 3 sessions were performed, and a random selection of 3 to 5 images was taken from each coverslip each session.

Following image acquisition, the fluorescence intensity was analyzed offline using Fiji v2.17.0 software. The fluorescence values are expressed as a percentage relative to the control value [30,49,63].

### 2.8. Evaluation of the Mitochondrial Inner Membrane Potential ΔΨ_m_ by Confocal Microscopy

Measuring the mitochondrial membrane potential (ΔΨ_m_) provides useful information about the general health of the mitochondria in living cells. Indeed, loss of ΔΨ_m_ is typically an upstream event in the apoptotic cascade, one leading to the opening of the mPTP and the release of cytochrome c [64].

ΔΨ_m_ was measured using the fluorescent probe tetramethylrhodamine ethyl ester (TMRE) at a concentration of 10 nM. This concentration allows the study of ΔΨ_m_ in nonquenching mode. Depolarized mitochondria (meaning mitochondria with lower ΔΨ_m_) would have lower concentrations of the cationic dye TMRE and thereby lower fluorescence, while polarized mitochondria (assumed to be healthy mitochondria) would have higher concentrations of TMRE and thus higher fluorescence [63,64,65,66].

Differentiated SH-SY5Y cells were seeded onto glass coverslips and later subjected to the experimental routine. Then, cells were loaded with 10 nM TMRE in the loading solution (30 min/37 °C) containing (in mM) 5 NaCl; 100 KCl; 70 mannitol; 25 sucrose; 10 KH2PO4; 10 Tris-HCl; 1 MgCl_2_; 5.5 glucose; 2 pyruvate; 2 malate; 3 succinate; and 0.1 ADP and buffered to pH 7.3 with KOH. Subsequently, cells were washed twice with the loading solution and loaded with 5 µM digitonin in the presence of 10 nM TMRE for 1 min. After wash-out, confocal images were acquired every 3 s with an inverted microscope Eclipse Ti2-E supplied with an AX confocal system (Nikon, Japan). Cells were excited at 543 nm and fluorescence was measured at 574 nm. Subsequently, FCCP (20 µM) was added to the loading solution and a dip in fluorescence was determined to certify the depolarization of the mitochondria.

Analysis of fluorescence intensity was performed offline after image acquisition. The TMRE fluorescence values were normalized to the trough values of FCCP exposure and are reported as percentages of the control value.

### 2.9. Immunofluorescence Staining of Aβ_1-42_ and pTau

SH-SY5Y cells and rat primary cortical neurons were severally plated onto 25 mm coverslips. These cells were subjected to the experimental protocol and then loaded with 300 nM MitoTracker (MitoTracker Red CMXRos M7512) (30 min/37 °C at 5% CO_2_). At the end, cells were fixed with 3.7% formaldehyde in PBS (30 min/RT). Subsequently cells were washed with glycine 0.1 M in PBS (5 min/4 °C) and permeabilized with 0.1% Triton-X100 in PBS (5 min/RT). Then, cells were incubated with Aβ_1-42_ (1:100) or pTau (1:1000) primary antibodies for 1.5 h. After washing the primary antibodies, cells were incubated with a secondary antibody (Anti-mouse Alexa Fluor 488 dye-conjugated antibody, dilution 1:200, Cat. A11059) for 30 min. Rat primary cortical neurons were loaded with Hoechst 33342 at a final concentration of 1 µg/mL (10 min/RT).

Confocal images were collected using a 510 LSM microscope with a META detection system (Carl Zeiss, Milan, Italy). Analysis of fluorescence intensity was performed offline, after image acquisition, using Fiji v2.17.0 software. The fluorescence values are expressed as percentages relative to the control values.

### 2.10. Detection of Cytosolic and ER Ca^2+^ Levels and SOCE

Ca^2+^ levels within cytosolic and ER compartments, as well as the SOCE response, were measured by single-cell computer-assisted videoimaging using an LSM 510 confocal system (Carl Zeiss, Milan, Italy). After being differentiated with RA into neuron-like cells and placed on 25 mm coverslips, SH-SY5Y cells underwent the experimental protocol. Subsequently, cells were loaded with 1 μM Fluo-4-AM in Krebs solution (45 min/37 °C) in the dark.

To measure cytosolic Ca^2+^ levels, basal fluorescence values were monitored for about 200 s, after which the Krebs solution was replaced with calibration solutions. Images were acquired every 5 s; the excitation light for Fluo-4-AM was provided by an argon laser at 488 nm and its fluorescence emission was recorded at 505–530 nm. The experiments were conducted with the following solutions: The Krebs solution contained (in mM) 140 NaCl; 5 KCl; 1 CaCl_2_; 0.5 MgCl_2_; 5.5 glucose; and 10 HEPES, buffered to pH 7.4 with NaOH. The Ca^2+^-free Krebs solution contained (in mM) 140 NaCl; 5 KCl; 0.5 MgCl_2_; 5.5 glucose; and 10 HEPES, buffered to pH 7.4 with NaOH. For calibration, two more solutions were prepared [63,67]:

Calibration solution 1 (0 Ca^2+^) was prepared by adding 0.3 M EGTA and 10 µM calcium ionophore (A23187) to the calcium-free Krebs solution.

Calibration solution 2 (high Ca^2+^) was prepared by adding 4 mM CaCl_2_ and 10 µM calcium ionophore (A23187) to the Ca^2+^-free Krebs solution.

Analysis of fluorescence intensity was performed offline after image acquisition. The fluorescence values are expressed as percentages relative to the control values.

ER Ca^2+^ levels and SOCE response were estimated by measuring the variations of cytosolic Ca^2+^ levels after exposure to the irreversible SERCA inhibitor thapsigargin (Tg) and a Ca^2+^ rich medium, respectively. After loading cells with 1 μM Fluo-4-AM, basal fluorescence was measured for about 150 s in a Ca^2+^-free solution. Then, 2 µM Tg was applied in a Ca^2+^-free solution for about 250 s. Finally, SOCE was identified by means of an increase in fluorescence after the application of a modified Krebs solution containing 3 mM Ca^2+^.

### 2.11. Evaluation of Mitochondrial Ca^2+^ Levels on Single Cells by Confocal Analysis

Basal Ca^2+^ levels within mitochondrial compartments were measured by single-cell computer-assisted videoimaging, as described above. Briefly, after being plated and differentiated onto 25 mm coverslips and undergoing the experimental protocol, SH-SY5Y cells were loaded with 2 μM Rhod-2-AM in DMEM (45 min/37 °C) in the dark, and subsequently rested in Krebs solution for 10 min in the dark. Basal fluorescence levels were measured for 200 s while cells were perfused with Krebs solution. Afterwards, the Krebs solution was replaced with calibration solution 1 until the lowest fluorescence value was stably reached (about 100 s); then, the calibration solution 1 (0 Ca^2+^ and EGTA) was replaced with calibration solution 2 (high Ca^2+^) to determine the highest fluorescent value. Images were acquired every 5 s using a 510 LSM microscope; the excitation light for Rhod-2-AM was provided by a helium–neon laser at 543 nm, and its fluorescence emission was recorded at 620 nm. At least 3 sessions were performed, in each of which at least 3 recordings were taken for each experimental group. Analysis of fluorescence intensity was performed offline after image acquisition. The fluorescence values are expressed as percentages relative to the control values [63].

### 2.12. Data Analysis

The data are expressed as mean ± S.E.M. The minimal level of significance was set to be *p* < 0.05. One-way ANOVA analysis followed by Dunnett’s post hoc test or the Holm–Sidak multiple comparisons test were used to calculate the differences between the mean values. The software used for the statistical analysis of the results was GraphPad Prism^®^ 5 (San Diego, CA, USA).

## 3. Results

### 3.1. BCH Restores ATP Synthesis and Cell Viability in SH-SY5Y Cells Exposed to GA

One of the earliest alterations in AD is a global neuronal hypometabolism, which feeds the progression of the neurodegenerative spiral in AD [15,68]. Given this, approaches aimed at restoring energy production and metabolic balance in neurons possess the potential to mitigate AD pathology expression [69,70].

**Figure 1:**
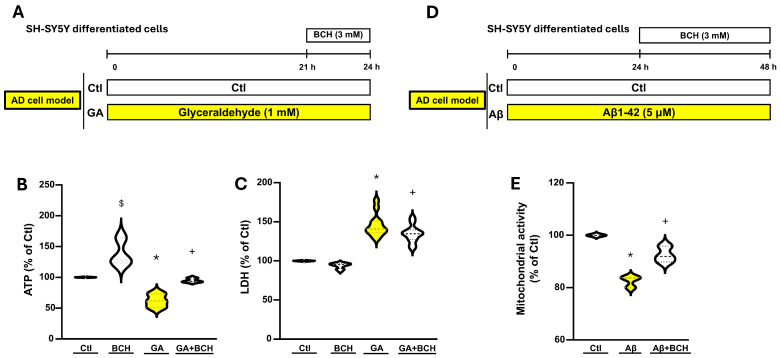
BCH-dependent GDH activation reverted GA-induced damage and Aβ1-42-induced mitochondrial dysfunction in RA-differentiated SH-SY5Y cells. (**A**) Timeline of the experimental protocol. For this AD model, differentiated SH-SY5Y cells were exposed to GA 1 mM for 24 h. From the 21st to the 24th h cells were treated with BCH 3 mM. At the end of the 24 h the experimental routines were carried out. (**B**) Violin graph depicting ATP values as measured by the luciferin–luciferase assay, in control conditions (Ctl), in control conditions with BCH (BCH 3 mM 3 h), after GA exposure (GA), and after both GA exposure and stimulation with BCH (GA + BCH 3 mM 3 h). The results are expressed as % of control. Each violin plot depicts the mean (indicated by the bold dashed line in the center) and the interquartile range (indicated by the fine dashed lines). Each plot groups data from 3 different experimental sessions. Differences among means were evaluated by one-way ANOVA followed by Dunnett’s post hoc test (* significant vs. control (*p* < 0.05); $ significant vs. control (*p* < 0.05); + significant vs. GA (*p* < 0.05) and BCH (*p* < 0.01)). (**C**) Violin graph depicting cell vitality as measured by the LDH assay, in control conditions (CTL), in control conditions with BCH (BCH 3 mM 3 h), after GA exposure (GA), and after both GA exposure and stimulation with BCH (GA + BCH 3 mM 3 h). The results are expressed as % of control. Each violin plot depicts the mean (indicated by the bold dashed line in the center) and the interquartile range (indicated by the fine dashed lines). Each plot groups data from 12 experiments from at least 3 different experimental sessions. Differences among means were evaluated by one-way ANOVA followed by Dunnett’s post hoc test (* significant vs. all groups (*p* < 0.01); + significant vs. all groups (*p* < 0.05)). (**D**) Timeline of the experimental protocol. For this AD model, differentiated SH-SY5Y cells were exposed to Aβ1-42 (Aβ, 5 μM/48 h). After 24 h from Aβ, cells were treated with BCH 3 mM for the remaining 24 h. At the end of the total 48 h the experimental routines were carried out. (**E**) Violin graph depicting mitochondrial activity measured by the MTT assay, in control conditions (Ctl), after Aβ1-42 exposure (Aβ), and after both Aβ1-42 and BCH (Aβ +BCH). The results are expressed as % of control. Each violin plot depicts the mean (indicated by the bold dashed line in the center) and the interquartile range (indicated by the fine dashed lines). Each plot groups data from at least 3 different experimental sessions. Differences among means were evaluated by one-way ANOVA followed by Dunnett’s post hoc test (* significant vs. all groups (*p* < 0.01); + significant vs. Aβ (*p* < 0.01)).

We decided to test whether BCH could improve cellular metabolism in our AD-like model of neurodegeneration. As reported in the timeline of the experimental protocol (Figure 1A), RA-differentiated SH-SY5Y cells were exposed to GA 1 mM for 24 h. Then, cells were treated with BCH 3 mM from the 21st to the 24th h of the GA exposure.

In these conditions, ATP cellular content was significantly reduced by the GA-exposure, but treatment with BCH (3 mM 3 h) managed to restore ATP to control levels (Figure 1A). Notably, BCH (3 mM 3 h) in control cells significantly increased ATP basal production. Moreover, as depicted in Figure 1C, GA increased LDH release while BCH prevented this detrimental effect (Figure 1C).

To strengthen the translational significance of these data, we decided to validate our findings in a more established AD model; therefore, we used amyloid-β oligomer-treated SH-SY5Y cells. Specifically, amyloid-β peptide 1-42 (Aβ1-42), prepared according to the manufacturer’s instruction, was applied to the cells at a final concentration of 5 µM for 48 h to induce Alzheimer’s disease–like cellular alterations. In the last 24 h of the treatment, RA-differentiated SH-SY5Y cells were exposed to BCH (see Figure 1D). In these conditions, mitochondrial activity was significantly reduced by Aβ1-42, but BCH (3 mM/24 h) managed to significantly restore this vital parameter (Figure 1E).

### 3.2. Treatment with BCH Reverts the Mitochondrial Damage Provoked by GA

**Figure 2:**
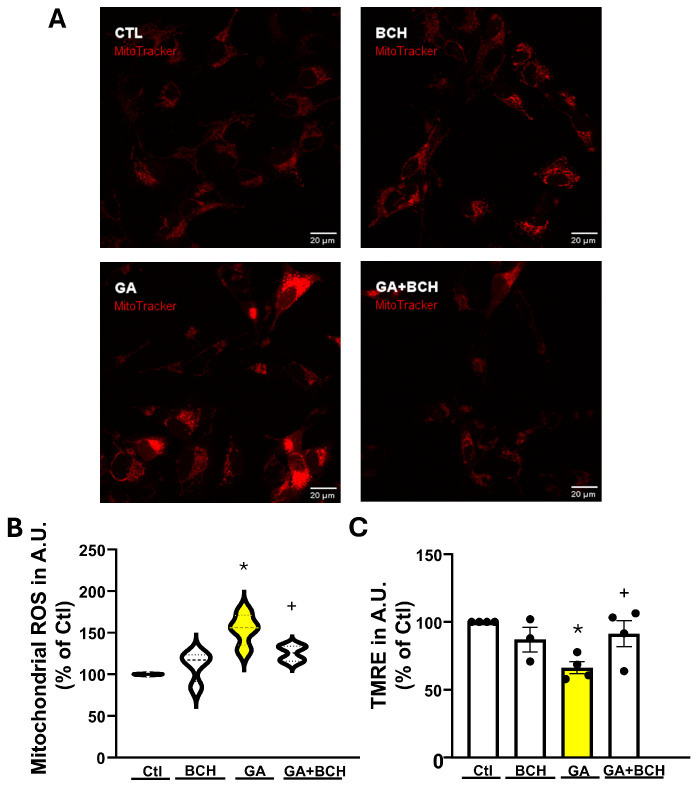
BCH-dependent GDH activation reverted GA-induced mitochondrial stress in RA-differentiated SH-SY5Y cells. (**A**) Representative images of mitochondrial ROS, as acquired by live cell imaging, in control conditions (CTL), in control conditions with BCH (BCH), after GA exposure (GA), and after GA + BCH. Red fluorescence represents MitoTracker CM-H2XRos. Scale bars: 20 µm. (**B**) Violin graph depicting the mitochondrial ROS quantification of CM-H2XRos fluorescence in control conditions (Ctl), in control conditions with BCH (BCH 3 mM 3 h), after GA exposure (GA), and after the combination of GA plus BCH (GA + BCH 3 mM 3 h). Results are expressed as % of control. Each violin plot depicts the mean (indicated by the bold dashed line in the center) and the interquartile range (indicated by the fine dashed lines). Each plot groups data from 5 different experimental sessions (at least 100 cells). Differences among means were evaluated by one-way ANOVA followed by Dunnett’s post hoc test (* significant vs. all groups (*p* < 0.05); + significant vs. GA (*p* < 0.05)). (**C**) Bar graph with dot overlay depicting the quantification of the mitochondrial membrane potential, as detected by TMRE in non-quenching mode, in control conditions (Ctl), in control conditions with BCH (BCH 3 mM 3 h), and after combination of GA plus BCH (GA + BCH 3 mM 3 h). Each bar plot depicts the mean and S.E.M. Results are expressed as % of control. Each bar groups data from at least 3 different experimental sessions (at least 100 cells). Differences among means were evaluated by one-way ANOVA followed by Dunnett’s post hoc test (* significant vs. CTL and GA + BCH (*p* < 0.05); + significant vs. GA (*p* < 0.05)).

Mitochondrial damage is a key component of AD neurodegeneration [15], and GA was capable of mimicking this facet of the disease in RA-differentiated SH-SY5Y cells by prompting a significant increase in mitochondrial ROS levels (Figure 2A). Therefore, we sought to investigate whether BCH might offer some protection against mitochondrial damage in our AD-like model.

Data obtained in single cells showed that GA induced a significant increase in mitochondrial ROS production that was significantly reduced by BCH (3 mM 3 h) exposure.

In order to assess the health status of the mitochondria, we decided to measure the mitochondrial membrane potential. Briefly, after we subjected cells to our protocol (as described in Figure 1), we loaded them with the TMRE dye, which is a positively charged dye that readily accumulates in active mitochondria.

As previously reported in rat cortical neurons challenged with GA [65], and as shown in Figure 2B, ΔΨ_m_ is severely affected by GA exposure; however, treatment with BCH restored the increased fluorescence levels to the levels associated with the control, indicating that the ΔΨ_m_ in the pharmacological treatment group was intact.

### 3.3. BCH Treatment Reverted GA-Induced Dysregulation of Calcium Homeostasis

Since Ca^2+^ dyshomeostasis is a central feature of AD neurodegeneration, we investigated the effects of BCH on calcium levels both in the cytosolic and mitochondrial compartment.

**Figure 3:**
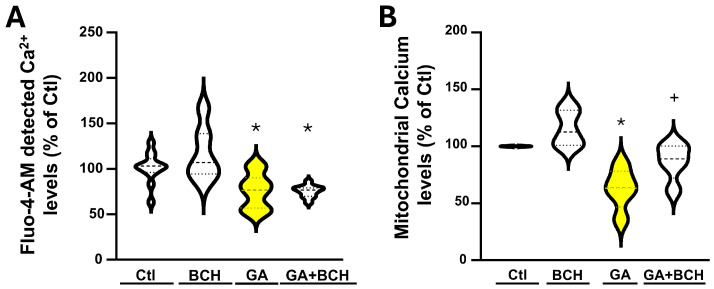
BCH-dependent GDH activation reverted GA-induced mitochondrial Ca^2+^ dyshomeostasis in RA-differentiated SH-SY5Y cells. (**A**) Violin graph depicting the quantification of cytosolic Ca^2+^, estimated by measuring Fluo-4-AM fluorescence in control conditions (CTL), in control conditions with BCH (BCH 3 mM 3 h), after GA exposure (GA), and after both GA exposure and stimulation with BCH (GA + BCH 3 mM 3 h). Results are expressed as % of control. Each violin plot depicts the mean (indicated by the bold dashed line in the center) and the interquartile range (indicated by the fine dashed lines). Each plot groups data from at least 5 different experimental sessions (at least 100 cells). Differences among means were evaluated by one-way ANOVA followed by Dunnett’s post hoc test (* significant vs. control (*p* < 0.05)). (**B**) Violin graph depicting the quantification of mitochondrial Ca^2+^, estimated by measuring Rhod-2-AM fluorescence in control conditions (CTL), in control conditions with BCH (BCH 3 mM 3 h), after GA exposure (GA), and after both GA exposure and stimulation with BCH (GA + BCH 3 mM 3 h). Results are expressed as % of control. Each violin plot depicts the mean (indicated by the bold dashed line in the center) and the interquartile range (indicated by the fine dashed lines). Each plot groups data from 5 different experimental sessions (at least 100 cells). Differences among means were evaluated by one-way ANOVA followed by Dunnett’s post hoc test (* significant vs. all groups (*p* < 0.05); + significant vs. GA and BCH (*p* < 0.05)).

As shown in Figure 3, GA exposure led to a reduction in Ca^2+^ levels in both the cytosol and mitochondria. Interestingly, BCH did not restore the cytosolic Ca^2+^ calcium levels disrupted by GA (Figure 3A), while it partially restored mitochondrial Ca^2+^ levels (Figure 3B).

**Figure 4:**
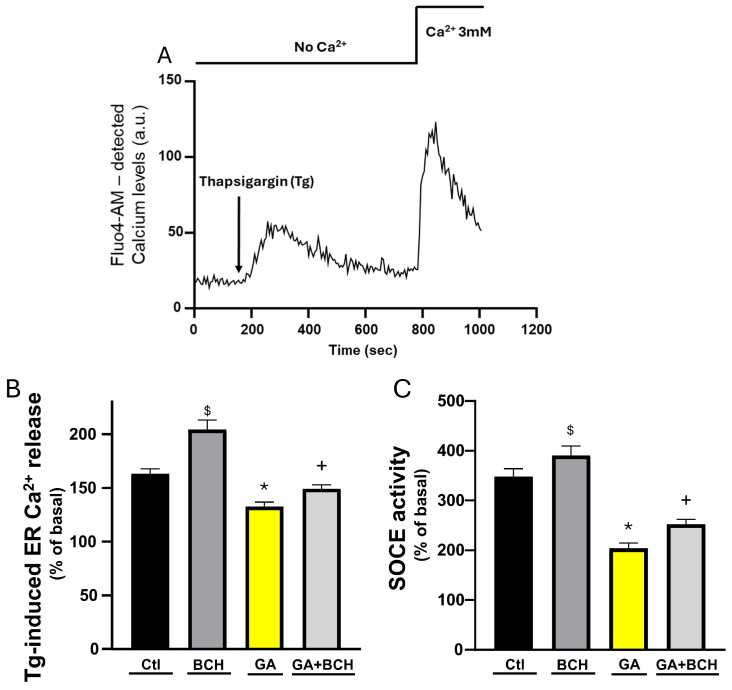
BCH-dependent GDH activation partially reverted GA-induced ER calcium dyshomeostasis in RA-differentiated SH-SY5Y cells. (**A**) Representative trace of Fluo-4-AM-detected Ca^2+^ in differentiated SH-SY5Y cells. Fluorescence was initially recorded in Ca^2+^-free-Krebs solution for 150 s, and cells were then exposed to Tg for about 600 s to measure ER- Ca^2+^-content and later perfused with a modified Krebs solution containing 3 mM Ca^2+^ to elicit SOCE. (**B**) Bar graph depicting ER Ca^2+^ content measured as Tg-induced Ca^2+^ increase in control conditions (Ctl), in control conditions with BCH (BCH 3 mM/3 h), after GA exposure (GA), and after both GA exposure and stimulation with BCH (GA + BCH 3 mM/3 h). Results are expressed as % of control. Each plot depicts the mean ± SEM. Each plot groups data from at least 3 different experimental sessions (at least 80 cells). Differences among means were evaluated by one-way ANOVA followed by the Holm–Sidak multiple comparisons test ($ significant vs. all other groups (*p* < 0.01); * significant vs. all other groups (*p* < 0.05); + significant vs. GA and BCH (*p* < 0.05)). (**C**) Bar graph depicting the quantification of SOCE as percentage of Ca^2+^ increase in response to Ca^2+^ reintroduction after Tg in Ca^2+^-free in control conditions (Ctl), in control conditions with BCH (BCH 3 mM/3 h), after GA exposure (GA), and after both GA exposure and stimulation with BCH (GA + BCH 3 mM/3 h). Results are expressed as % of control. Each plot depicts the mean ± SEM. Each plot groups data from at least 3 different experimental sessions (at least 80 cells). Differences among means were evaluated by one-way ANOVA followed by Dunnett’s post hoc test ($ significant vs. GA and GA + BCH (*p* < 0.01); * significant vs. all other groups (*p* < 0.05); + significant vs. all other groups (*p* < 0.05)).

In the determination of the effects of GA on cytosolic and mitochondrial Ca^2+^ levels, the involvement of other Ca^2+^ stores in the metabolic damage caused by GA has also been addressed. To this end, we measured both the ER Ca^2+^ levels and SOCE as Ca^2+^ increased after the addition of the irreversible SERCA inhibitor Tg, and subsequent to the sequent reintroduction of 3 mM Ca^2+^, respectively (Figure 4A).

Indeed, as shown in Figure 4B, the ER calcium store was significantly depleted by GA exposure and BCH managed to partly restore it. Moreover, BCH partially restored the SOCE response, which was severely compromised by GA (Figure 4C).

### 3.4. Treatment with BCH Completely Prevented GA-Induced Increase in pTau and Aβ_1-42_ Expression as Measured with Immunocytochemistry

In recent years, there have been a number of advancements in diagnostic methodologies for AD, such as the emergence of new biomarkers [71] and the development of blood-based markers, making the biological diagnosis of AD much more accessible [72]. However, in the Revised criteria for diagnosis and staging of Alzheimer’s disease of the Alzheimer’s Association, β-amyloid and pTau are still regarded as basic biomarkers for the diagnosis of AD [72].

The interplay between oxidative stress, mitochondrial dysfunctions, Ca^2+^ dyshomeostasis and pTau and Aβ expression has been extensively investigated [73,74], and it is already established that GA can induce an increase in the levels of both pTau and Aβ in SH-SY5Y cells [63].

Therefore, we decided to investigate whether the treatment with BCH 3 mM in the last 3 h of the GA challenge could lower pTau and Aβ levels, as measured with immunocytochemistry. In particular, double immunocytochemistry has been performed using MitoTracker Red CMXRos and antibodies against each AD aggregated protein.

**Figure 5:**
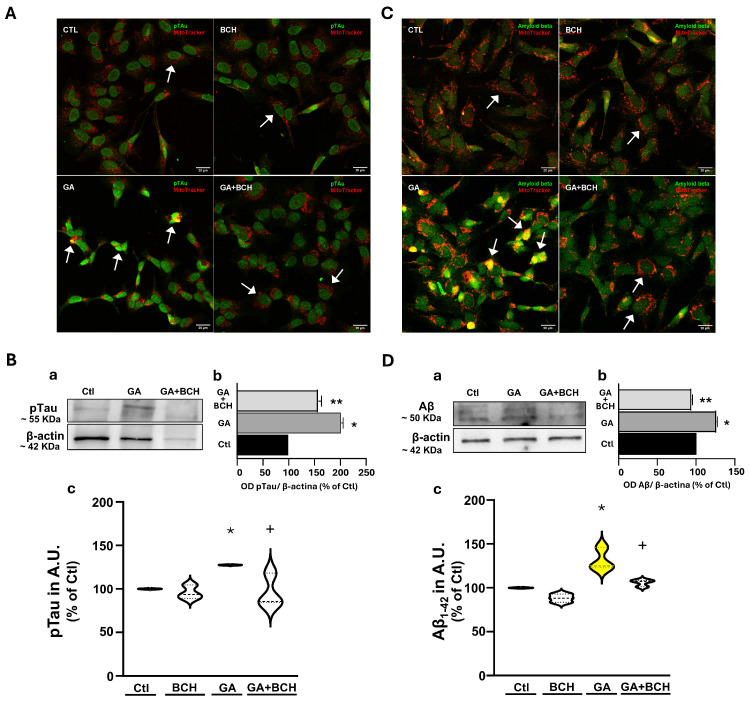
BCH-dependent GDH activation completely reverted GA-induced pTau and Aβ_1-42_ accumulation in RA-differentiated SH-SY5Y cells. Cells were exposed to GA 1 mM for 24 h. From the 21st to the 24th h cells were treated with BCH 3 mM. (**A**) Representative images of pTau, acquired by immunofluorescence. Green is pTau, red is MitoTracker Red CMXRos. Scale bars: 20 µm. Arrows indicate representative cells, showing higher concentration of pTau in the GA group. (**B**) Quantification of pTau as measured by Western blotting (**a**,**b**) and immunofluoresce staining (**c**). Results are expressed as % of control. (**a**,**b**) Each bar depicts the mean of 3 different experiments (* significant vs. Ctl (*p* < 0.0001), ** significant vs. GA (*p* < 0.001)). (**c**) Each violin plot depicts the mean (indicated by the bold dashed line in the center) and the interquartile range (indicated by the fine dashed lines). Each plot groups data from 3 different experimental sessions (at least 100 cells). Differences among means were evaluated by one-way ANOVA followed by Dunnett’s post hoc test (* significant vs. all groups (*p* < 0.05); + significant vs. GA (*p* < 0.05)). (**C**) Representative images of Aβ1-42, acquired by immunofluorescence. Green is Aβ1-42, red is MitoTracker Red CMXRos. Scale bars: 20 µm. Arrows indicate representative cells, showing higher concentration of Aβ_1-42_ in the GA group. (**D**) Quantification of Aβ_1-42_ as measured by Western blotting (**a**,**b**) and immunofluoresce staining (**c**). Results are expressed as % of control. (**a**,**b**) Each bar depicts the mean of 3 different experiments (* significant vs. Ctl (*p* < 0.0001), ** significant vs. GA (*p* < 0.0001)). (**c**) Each violin plot depicts the mean (indicated by the bold dashed line in the center) and the interquartile range (indicated by the fine dashed lines). Each plot groups data from 3 different experimental sessions (at least 100 cells). Differences among means were evaluated by one-way ANOVA followed by Dunnett’s post hoc test (* significant vs. all groups (*p* < 0.01); + significant vs. GA (*p* < 0.01)). Original images of (**B**,**D**) can be found in Supplementary Materials.

As shown in Figure 5, GA led to significant increases in the immunosignals of both pTau (Figure 5A,B) and Aβ1-42 (Figure 5C,D), which colocalized with mitochondria, while treatment with BCH managed to partially restore the levels of both biomarkers. The same results were obtained by Western blotting analysis (see insets a,b of Figure 5B,D), showing the ability of BCH (3 mM) to restore pTau and Aβ1-42 expression levels altered by GA.

### 3.5. Treatment with BCH Restores ATP Synthesis, Cell Viability and Biomarkers Levels in Primary Rat Cortical Neurons Exposed to GA

To strengthen the robustness of our previous results, we decided to replicate some of the more relevant and fundamental experiments in rat primary cortical neurons. The neurons were subjected to the same protocol as SH-SY5Y, as detailed in Figure 1. Briefly, they were exposed to GA 1 mM for 24 h in order to induce alterations typically observed in AD neurons [57,63]. From the 21st to the 24th h of GA exposure, cells were treated with BCH 3 mM.

**Figure 6:**
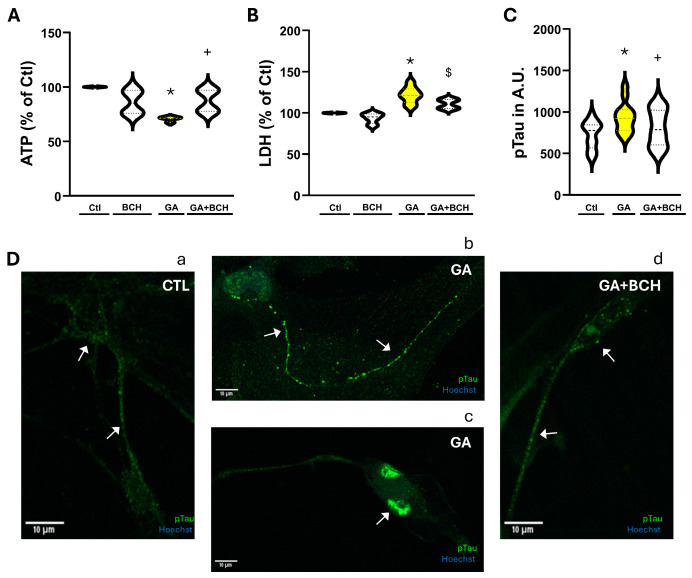
BCH-dependent GDH activation reverted GA-induced damage in rat cortical neurons (DIV 9). Neurons were exposed to GA 1 mM for 24 h. From the 21st to the 24th h cells were treated with BCH 3 mM. (**A**) Violin graph depicting ATP values as measured by the luciferin–luciferase assay in control conditions (CTL), in control conditions with BCH (BCH 3 mM 3 h), after GA exposure (GA), and after both GA exposure and stimulation with BCH (GA + BCH 3 mM 3 h). Results are expressed as % of control. Each violin plot depicts the mean (indicated by the bold dashed line in the center) and the interquartile range (indicated by the fine dashed lines). Each plot groups data from 4 experiments performed in 3 different sessions. Differences among means were evaluated by one-way ANOVA followed by Dunnett’s post hoc test (* significant vs. control (*p* < 0.01); + significant vs. GA (*p* < 0.05)). (**B**) Violin graph depicting cell vitality, as measured by the LDH assay in control conditions (CTL), in control conditions with BCH (BCH 3 mM 3 h), after GA exposure (GA), and after both GA exposure and stimulation with BCH (GA + BCH 3 mM 3 h). Results are expressed as % of control. Each violin plot depicts the mean (indicated by the bold dashed line in the center) and the interquartile range (indicated by the fine dashed lines). Each plot groups data from at least 4 experiments performed in 3 different sessions. Differences among means were evaluated by one-way ANOVA followed by Dunnett’s post hoc test (* significant vs. all other groups (*p* < 0.05); $ significant vs. all other groups (*p* < 0.05)). (**C**) Quantification of pTau as measured by immunofluorescence staining. Results are expressed as arbitrary units. Each violin plot depicts the mean (indicated by the bold dashed line in the center) and the interquartile range (indicated by the fine dashed lines). Each plot groups data from 3 different experimental sessions (at least 20 cells). Differences among means were evaluated by one-way ANOVA followed by Dunnett’s post hoc test (* significant vs. all groups (*p* < 0.05); +significant vs. GA (*p* < 0.05)). (**D**) Representative images of pTau, acquired by immunofluorescence, in control conditions (**a**), after GA-exposure (**b**,**c**) and after GA exposure and BCH pharmacological treatment (**d**). Green is pTau, blue is Hoechst. Scale bars: 10 µm. Arrows indicate representative cells, showing aggregates and higher concentration of pTau in the GA group.

In accord with data observed in SH-SY5Y cells, we measured significant reductions in both ATP cellular content (Figure 6A) and cell vitality (Figure 6B) in rat primary cortical neurons exposed to GA. However, treatment with BCH completely restored the ATP levels to the level associated with the control (Figure 6A) and managed to mitigate the GA-induced damage, as evidenced by the LDH assay (Figure 6B).

Interestingly, GA induced an accumulation of pTau aggregates in both soma and processes distributed as a dot-like immunosignal (Figure 6C,D). However, treatment with BCH prevented AD-related detrimental accumulation in both cellular domains (Figure 6C,D), thus further validating the GA model and the identification of a putative new pharmacological approach.

## 4. Discussion

Metabolic alterations emerge years before the onset of AD clinical manifestations, characterizing a hypometabolism in which energy failure, oxidative stress, ionic dyshomeostasis and protein accumulation are intimately connected [15,16]. In this context, BCH proved to be able to stimulate energy production in physiological and pathological conditions, likely by fostering the TCA cycle via the conversion of glutamate into α-ketoglutarate, thereby promoting the synthesis of ATP and replenishing the cellular energy reserves.

Accordingly, in this study, we showed that allosteric activation of GDH by BCH in our AD-like models was able to increase ATP cellular content and ΔΨ_m_, while also reducing LDH release, ROS production, pTau and Aβ_1-42_ levels.

Mechanistically, the pharmacological stimulation of GDH by BCH likely restores the cell reserves of reduced NADH in two ways. Firstly, the reductive amination of glutamate into α-ketoglutarate leads to the reduction of NADH as a byproduct. Secondly, when the new α-ketoglutarate undergoes the TCA cycle, it promotes the formation of 3 NADH. NADH is then capable of entering the electron transport chain, where it donates electrons to pump protons to the mitochondrial intermembrane space, keeping the mitochondrial inner membrane potential. The proton gradient is then used by the ATP synthase to generate ATP, thereby leading to an increase in ATP cellular content. Since a low ΔΨ_m_ leads to inefficient OXPHOS, the rate of ROS formation increases, leading to oxidative stress. Oxidative stress, in turn, damages a vast range of mitochondrial enzymes, including the electron transport chain (Rummel et al., 2022) [75] and several transporters essential for maintaining ionic homeostasis. This mechanism feeds a positive (but vicious) feedback cycle, promoting oxidative stress and damage to mitochondria, ultimately leading to the opening of the MCU and the release of cytochrome c and eventually, to apoptosis. By increasing the formation of NADH and thereby preventing the loss of ΔΨ_m_, BCH manages to suppress the formation of excessive ROS, protecting mitochondria from oxidative stress and mitigating cell death. Furthermore, the refilling of the TCA cycle with α-ketoglutarate promotes the formation of fumarate, a TCA intermediate capable of activating the glutathione peroxidase 1, which is one of the most effective antioxidant defenses. This could be another mechanism through which BCH suppresses oxidative stress (Jin et al., 2015) [76]. Moreover, as previously described, GDH has a complex regulation involving, among other factors, the levels of NADH. Therefore, in looking for a reason, the lack of a ΔΨ_m_ boost in the BCH control group might be ascribed to the inhibitory effect of the physiological levels of NADH in mitochondria on the activity of GDH. On the other hand, the low concentration used (3 mM) limited off-target effects [75,76,77,78].

Furthermore, our results indicate that GA-provoked neurodegeneration causes a profound Ca^2+^ dyshomeostasis, affecting various cellular compartments, such as mitochondria, cytosol and ER. Moreover, cellular responses tasked with the regulation of Ca^2+^ storing—i.e., SOCE, which is typically impaired in AD neurons [24,26]—are severely affected as well, adding another point of parallelism between AD and our GA model. Although BCH did not manage to completely reverse said alterations, it boosted the SOCE response, likely via an increase in energy availability, which likely allowed the ER to restore its calcium gradient. Notably, treatment with BCH normalized mitochondrial calcium levels, suggesting another facet of mitochondrial protection by BCH against the GA metabolic damage to the already discussed effects on mitochondrial membrane potential and oxidative stress. To elaborate, mitochondria act as Ca^2+^ buffers thanks to the electrochemical gradient generated by the ETC, which produces a membrane potential that allows the transport of calcium into the mitochondrial matrix [24]. It is likely that by stabilizing the ΔΨm, BCH allowed Ca^2+^ to move inside the mitochondria, which would explain the normalization of the mitochondrial calcium levels registered in the BCH treatment group.

It is important to mention the occurrence of the functional cooperation between mitochondria and ER in handling intracellular Ca^2+^. In this context, ER Ca^2+^ content in the steady state is the result of an equilibrium between the activity of SERCA, which pumps Ca^2+^ inside the ER; the physiological leaking of Ca^2+^ from the ER membrane; and the exchange of Ca^2+^ with other organelles, including mitochondria [79] However, in AD, ryanodine receptors (RyRs) have been observed to leak a larger quantity of Ca^2+^ from the ER and Aβ has been shown to stimulate the release of calcium from RyRs (Lacampagne et al., 2017) [80]. This observation, which is in agreement with our own measurements of ER Ca^2+^ levels, could explain the low efficacy of BCH in totally restoring ER Ca^2+^ levels, since the higher ATP availability might be made futile by an ineffective cycle, one in which calcium is pumped by SERCA, while hyperactive RyRs determine a high efflux from the ER. In this respect, the action of BCH should be enhanced by an effective inhibition of RyRs or a potentiation of refilling mechanisms (SERCA and SOCE). Further investigations are needed to clarify the complexity of calcium regulation under conditions of GA-induced stress and the roles of other cellular compartments. For example, lysosomes communicate continuously with the ER via the interaction between STIM1 and TRPML1, regulating lysosomal and ER Ca^2+^ levels. Notably, these targets (and thereby their interactions) are severely impaired in many pathological and neurodegenerative conditions [27,28,29].

Moreover, while the protective effects of BCH likely stem from the increase in energy availability in the cell and the normalization of the mitochondrial membrane potential, they reverberate throughout the whole cellular metabolism. Of note, ROS have been shown to activate GSK3β and cyclin-dependent protein kinase-5 (CDK5), both responsible for the hyperphosphorylation of tau, while also causing the formation of disulfide bonds in tau, which promotes its aggregation [21]. Furthermore, oxidative stress has been shown to influence the fate of APP and regulate the activity of secretases, specifically, increasing the expression of APP, while promoting BACE1 enzymatic function, and thus prompting reduced levels of full-length APP and the non-amyloidogenic sAPPα, and ultimately promoting Aβ aggregation [21]. Therefore, the reduction in mitochondrial ROS production provoked by BCH shifts the processing of APP from the amyloidogenic to the non-amyloidogenic pathway and reduces the hyperphosphorylation of tau, thereby resulting in sharp reductions in the levels of biomarkers.

All the previously discussed cellular phenomena ultimately converge in the increase in cell vitality observed. Of course, although our results highlight benefits in stimulating GDH, this avenue should also be explored in models more closely recapitulating the complexities of the human AD brain, including animal models of AD.

## 5. Conclusions

In conclusion, our work offers a proof of concept for a therapeutic approach targeting glutamate metabolism via the activation of GDH, aiming to restore multiple pathological features associated with AD-related neurodegeneration.

Indeed, our GA model recapitulates several key pathognomonic findings typical of the AD neurodegenerative spiral, such as oxidative stress, the reduction of the mitochondrial membrane potential and the rise in AD biomarkers.

Within this intricate network of neurodegeneration, BCH was capable of fostering energy production via the pharmacological activation of GDH and the subsequent conversion of glutamate into α-ketoglutarate. The refilling of the TCA cycle by the newly formed α-ketoglutarate boosted OXPHOS, increasing ATP production. This increased energy reserve allowed the cell to better face the GA metabolic damage. Moreover, the NADH produced in the conversion of glutamate into α-ketoglutarate likely improved the cell’s ability to retain the mitochondrial membrane potential by carrying high-energy electrons to the ETC to be used to pump protons in the intermembrane space. The physiological mitochondrial membrane potential allows mitochondria to function correctly, abolishing the increased ROS formation caused by the GA-induced neurodegenerative damage, and subsequently reducing the expression of AD biomarkers. Moreover, an increased energy supply enables the cell to recover ion gradients, allowing it to partially restore mitochondrial and ER calcium levels and the SOCE response.

All the above-described mechanisms ultimately resulted in a protective response of BCH in terms of cell survival and biomarkers levels (i.e., pTau and Aβ). The same effect was produced in a more established model of AD consisting of amyloid-β oligomer–treated SH-SY5Y cells. Also in this model, BCH significantly restored Aβ1-42-induced mitochondrial activity reduction.

We believe these results to be forerunners to approaches based on the targeting of alternative metabolic pathways, which would enable neurons to face the metabolic challenges of neurodegenerative diseases like AD.

## Data Availability

Data will be made available on request.

## Supplementary Materials

The following supporting information can be downloaded at: https://www.mdpi.com/article/10.3390/biom16050667/s1, Original images of Figure 5B,D can be found in Supplementary Materials.
