# Supplementary figures and images for: Allosteric Activation of GDH/TCA Pathway Reduces Pathological Build-Up and Promotes Neuronal Survival in an In Vitro Model of Alzheimer’s Disease
Source: Biomolecules. 2026 Apr 30;16(5):667. doi: 10.3390/biom16050667 (PMC13204450; doi:10.3390/biom16050667)

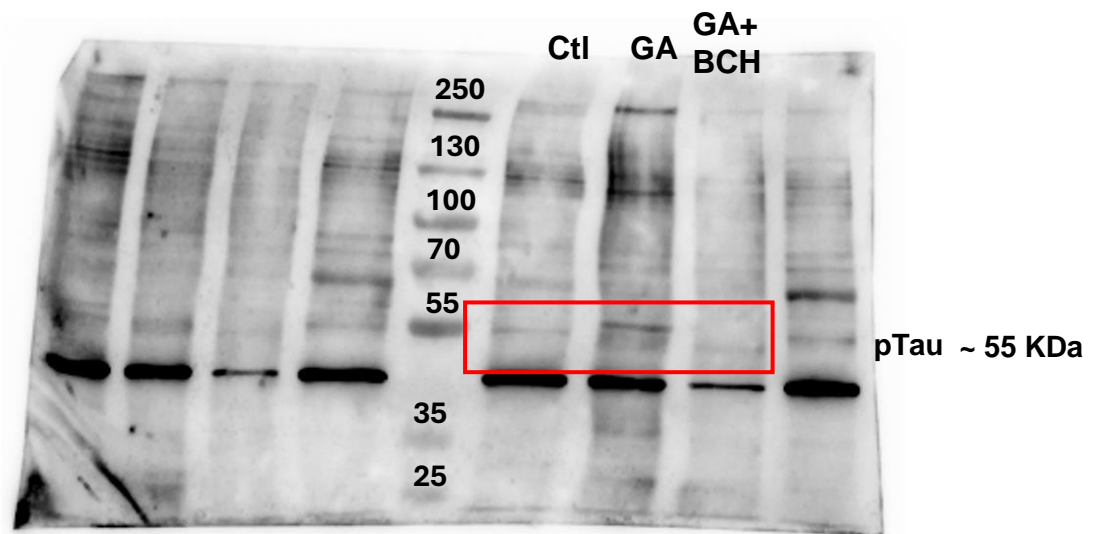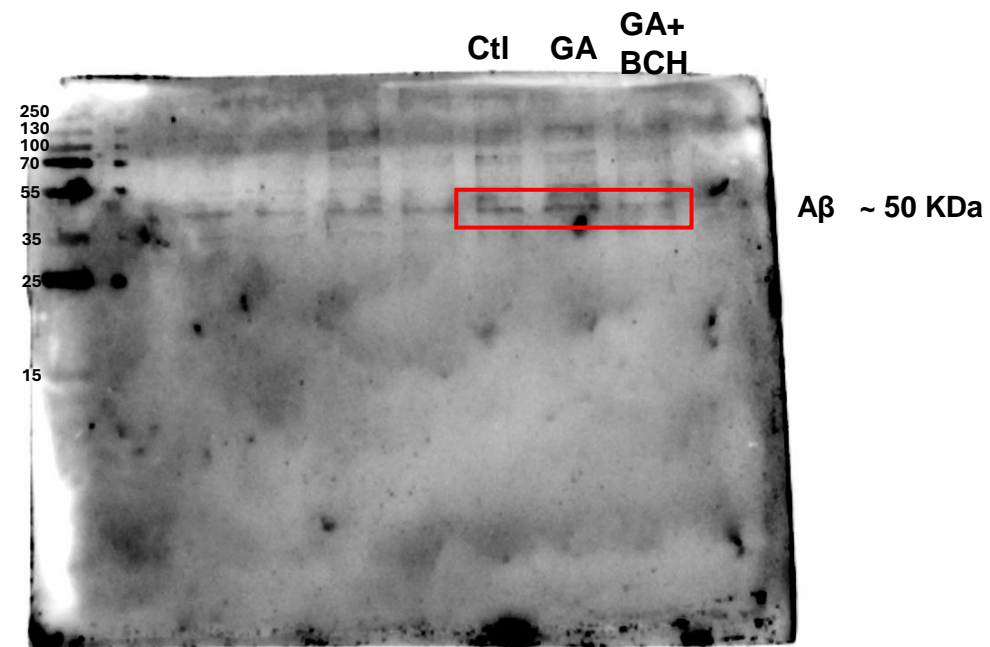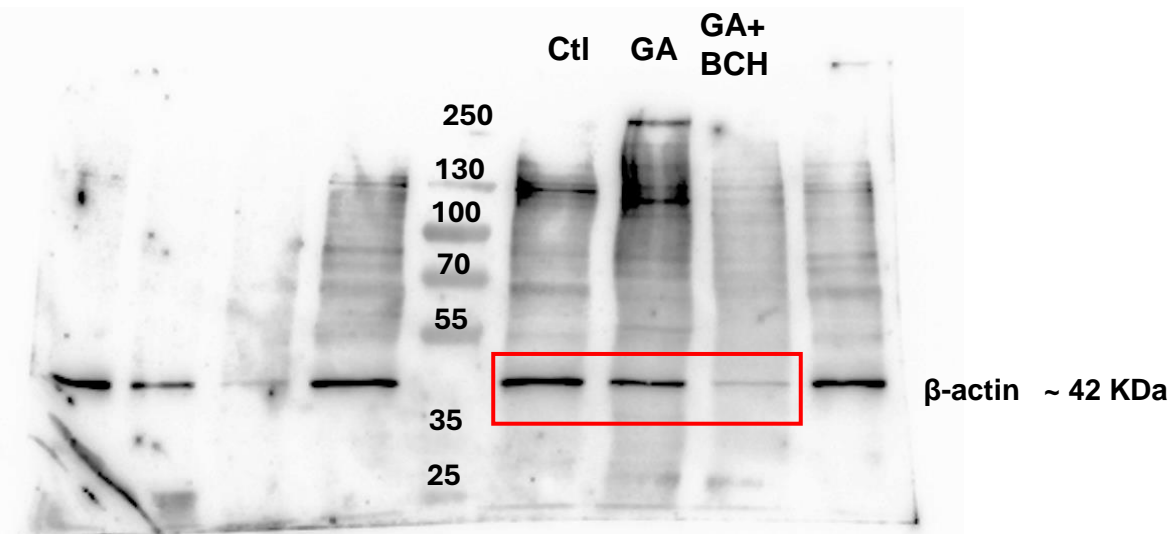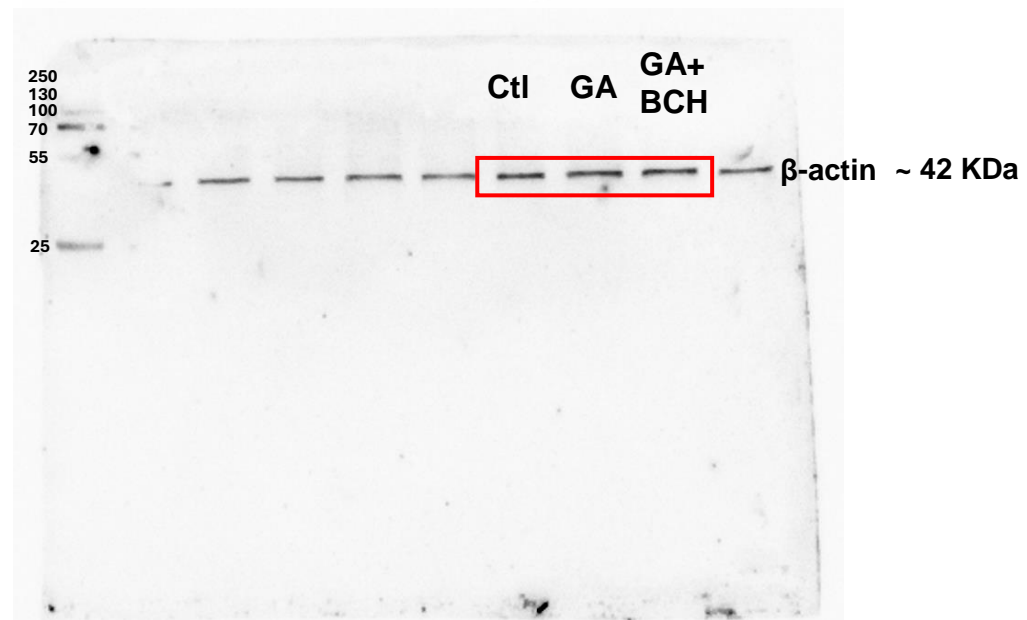

Supplement: Supplementary file 1 [file biomolecules-16-00667-s001.zip › biomolecules-4253864-supplementary.pdf]
